# Physicochemical, Functional, Fatty Acids Profile, Health Lipid Indices, Microstructure and Sensory Characteristics of Walnut-Processed Cheeses

**DOI:** 10.3390/foods10102274

**Published:** 2021-09-26

**Authors:** Khaled A. Abbas, Hani S. Abdelmontaleb, Shaimaa M. Hamdy, Abderrahmane Aït-Kaddour

**Affiliations:** 1 Dairy Department, Faculty of Agriculture, Fayoum University, Fayoum 63511, Egypt; hsm00@fayoum.edu.eg (H.S.A.); smh01@fayoum.edu.eg (S.M.H.); 2 VetAgro Sup, INRAE (National Institute for Agriculture, Food, and Environment), Université Clermont Auvergne, UMRF, 89 Avenue de L’Europe, F-63370 Lempdes, France; abderrahmane.aitkaddour@vetagro-sup.fr

**Keywords:** functional processed cheese, omega fatty acids, walnuts, atherogenic index, thrombogenic index

## Abstract

In the present study, processed cheeses fortified with walnut paste (a high source of omega-3 fatty acids) were developed and characterized. In order to identify the best cheese formulation, the effects of different proportions of walnut paste (0, 5, 10, and 15%) on cheese physicochemical, functional, fatty acids profile, health lipid indices (atherogenic and thrombogenic), microstructure, and sensorial characteristics were studied. Results showed that walnut-added samples had significantly (*p ≤* 0.05) higher levels of acidity, protein, fat, and ash contents with lower meltability and oil separation index compared to the control. Processed cheeses with walnuts contained significantly (*p ≤* 0.05) higher percentages of MUFAs, and *ω*-3 PUFAs (mainly α-linolenic acid) and significantly (*p* ≤ 0.05) lower amounts of SFAs (mainly myristic, palmitic and stearic acids) and *ω*_6_/*ω*_3_ ratio. Scanning Electron Micrograph of processed cheese containing walnut paste showed uniform distribution of walnut in the protein matrix. Processed cheeses made with 5 or 10% walnut paste presented the most acceptable sensory properties. These results indicated that walnut paste supplementation can be used as a nutritional strategy to increase concentrations of human health-promoting fatty acids in processed cheeses while maintaining good sensory and technological properties.

## 1. Introduction

Food technologists and nutritionists are making great efforts to develop healthy and nutritious foods. One of the strategies is to incorporate nuts, fruits, vegetables, herbs, and spices in the formulation of processed food [1]. Among nuts, walnuts (*Juglans regia*) are one of the richest sources of unsaturated fatty acids [2,3]. The walnut contains high levels of long-chain polyunsaturated fatty acids (PUFAs), especially linoleic (*ω*_6_ PUFAs) and α-linolenic acid (*ω*_3_ PUFAs), that have a beneficial effect on plasma lipid profiles and reduce the risk of cardiovascular disease, coronary heart disease, diabetes, and some cancer types [4,5,6]. Walnuts are utilized in the food industry as an ingredient in the production of biscuits, bread, cakes, and as a functional additive in meat products [7]. One of the most important strategies to augment the presence of walnuts in the diet could be to incorporate them into frequently consumed products like processed cheeses.

Processed cheeses are consumed for their high nutritional value and are more widely accepted by the younger consumer due to their diversity of sensory characteristics such as flavour and texture. Therefore, it is extensively used in many applications in both the raw and the heated forms in the fast-food/catering chains. It is made from blending and melting one or more natural cheese types, water, emulsifying salt, and optional ingredients (dairy or non-dairy) with the aid of heat (80–95 °C/5–10 min) and with a constant stirring to form a molten homogeneous mass [8]. Processed cheese offers excellent ways to promote the intake of functional compounds (*ω*_3_ fatty acids) without any changes in eating habits. The development of functional processed cheese has been intensively studied in recent years in various ways in the view of increasing their health benefits effects [9,10,11,12,13,14,15,16]. As far as we know, no studies have been reported to incorporate walnut paste in processed cheese formulations to improve their fatty acids profile and related health lipids indices.

Therefore, the present study aimed to investigate the effect of walnut paste (0, 5, 10, and 15%) on the physicochemical, fatty acid profile and related health lipids indices, microstructure, and sensory characteristics of processed cheeses.

## 2. Materials and Methods

### 2.1. Materials

A 4 kg block of commercial Edam cheese matured for 3 months (average composition: 45% moisture, 23% protein, 28% fat, and 3.5% ash) was cut into small pieces, and used for the production of processed cheeses. Walnuts (1 kg) were purchased in the roasted state from an Egyptian local market and were ground intensively for 2 min using a kitchen food processor (Samsung, Seoul, South Korea) in order to obtain uniform walnuts and were ground and underwent a second roasting at 150 °C for 20 min in oven to obtain a sterilized walnut paste (3.89% moisture, 15.31% protein, 61.67% fat, and 2.02% ash) and this paste was directly applied in cheese formulations with appropriate ratio. Trisodium citrate (Sigma-Aldrich Chemie Gmbh, Munich, Germany) was used as emulsifying salts.

Walnuts were purchased in a roasted state.

### 2.2. Methods

#### 2.2.1. Formulation and Processing of Walnuts Processed Cheese

Four different processed cheese formulations containing different ratios of walnut paste were prepared using Edam cheese (made from cow milk) as a base blend as described in Rafiq and Ghosh [11]. Briefly, the milled Edam cheese was added to a pilot-scale cheese cooker (Stephan Machinery Corp., New York, NY, USA) and mixed with water (10–20% of the cheese weight), and emulsifying salt (3%). The mixture was heated at 85–90 °C for 5 min with continuous stirring (1500 rpm) in the cheese cooker until the cheese mass became a homogenous mass. Walnut paste was added to this basic mixture at ratios (0, 5%, 10, and 15% *w*/*w*) and then each of formulations was cooked for 5 min at 75–80 °C with continuous mixing. The hot molten cheese was filled individually into 250 cm^3^ plastic containers, cooled for 2 h at room temperature, and then placed in a laboratory refrigerator (4 ± 1 °C) for further analysis.

#### 2.2.2. Physicochemical and Functional Analysis

Titratable acidity, moisture, fat, ash, and protein contents of the processed cheese samples were determined in triplicate by A.O.A.C. [17] methods. Measurement of pH was carried out using a pH meter (Kent EIL 7020).

Meltability of processed cheese samples was measured using a Schreiber test, with the following modifications: three discs (4 cm diameter, 10 mm thickness) from each cheese sample were placed in a glass Petri dish and heated in an air-circulated oven at 110 °C for 10 min, and then cooled for 15 min at room temperature. Meltability (%) was expressed as a ratio of (area of molted disc–area of the original disc)/area of original disc × 100 [18]. The oil separation index was determined as suggested by Thomas [19], three discs of each sample were placed onto Whatman No. 41 filter paper and then placed into an oven at 110 °C for 10 min. The fat soaks into the filter paper and forms a grease ring around the melted cheese disc. The oil separation (%) was calculated as follows: (area of fat ring and disc–area of original disc)/area of original × 100. Meltability and oil separation index were given as the mean of three readings for each treatment.

#### 2.2.3. Fatty Acids Profile

Free fatty acids (FFA) profile of processed cheese samples was determined according to Maguire, O’sullivan, Galvin, O’connor and O’brien [20] using gas chromatography-mass spectrometry instrument (TRACE Ultra Gas Chromatographs, THERMO Scientific Corp., Santa Clara, CA, USA), coupled with a thermos mass spectrometer detector (ISQ Single Quadrupole Mass Spectrometer). Analyses were carried out using helium as carrier gas at a flow rate of 1.0 mL/min and a split ratio of 1:10 using the following temperature program: 60 °C for 1 min; rising at 4 °C/min to 240 °C and held for 1 min. The injector and detector were held at 210 °C. Diluted samples (1:10 hexane) of 1 μL of the mixtures were injected, and the run time was 10 min. Mass spectra were obtained by electron ionization at 70 eV, using a spectral range of m/z 40–450. The identification of the chemical constituents of the walnut oil was de-convoluted using AMDIS software and identified by its retention indices (relative to n-alkanes C6-C22).

#### 2.2.4. Health Lipid Indices

Health lipids indices of processed cheeses were assessed using different combinations and ratios between fatty acids such as total saturated fatty acids (ΣSFA), total monounsaturated fatty acids (ΣMUFA), total polyunsaturated fatty acids (ΣPUFA), PUFA/SFA and *ω*-6/*ω*-3 ratios. In addition, desirable fatty acids (DFA), atherogenic index (AI), and thrombogenic index (TI) were calculated according to Ahmad et al. [21] using Equations (1)–(3), respectively.

DFA = ΣUFA + C18:0(1)

AI = (C12:0 + 4*C14:0 + C16:0)/(*ω*-_3_ PUFA + *ω*-_6_ PUFA + MUFA) (2)

TI = (C14:0 + C16:0 + C18:0)/(0.5MUFA + 0.5 *ω*-_6_ PUFA + 3 *ω*-_3_PUFA + *ω*-_3_/*ω*-_6_) (3)

#### 2.2.5. Cheese Microstructure

Scanning electron microscopy (SEM) was used to evaluate the microstructure of the processed cheese samples. The samples were prepared using the method described by Cunha, Dias and Viotto [22]. Fixed and dehydrated samples were dried in a CPD 030 critical point dryer (Bal-tec AG, Balzers, Liechtenstein), fractured at room temperature, mounted on aluminum stubs with silver glue, and observed under a scanning electron microscope (JEOL-USA, Inc., Peabody, MA, USA) at 10 kV. Representative SEM micrographs were selected for presentation. Images were presented at 500× and 1000× magnifications.

#### 2.2.6. Sensory Evaluation

Processed cheese samples were organoleptically evaluated for appearance, texture, taste, smell, and overall acceptability at room temperature by 10 untrained panelists (staff and students, 6 men and 4 women, aged from 20 to 45 years) familiar with processed cheese. The 10 assessors (university professors and postgraduate students) were chosen based on their perfect knowledge of the sensory analysis and characteristics of good quality dairy products especially cheese. They were also chosen because they consume frequently processed cheese, at least three times a week. Samples were presented to panelists in white plastic cups under natural room light. The evaluation process was performed after 1 day of manufacture using a nine-point hedonic scale on which 1 indicated “dislike extremely” and 9 indicated “like extremely” [23]. The overall acceptability score was calculated as the sum of scores of all the sensory attributes. Water was provided to rinse the mouth (to cleanse the palate) after each sample evaluation.

#### 2.2.7. Statistical Analysis

All analyses were carried out in triplicate and obtained results were expressed as mean values ± standard deviation. The difference among the mean values was statistically evaluated by using one-way analysis of variance (ANOVA) through LSD test (*p* ≤ 0.05) using XLSTAT Statistical software version 2007 (Addinsoft, Paris, France). Principal Component Analysis (PCA) was performed on physicochemical and functional data by MATLAB R2012b software (The MathWork, Natick, MA, USA) coupled with the PLS Toolbox v.7.5 (Eigenvector Research, Manson, WA, USA).

## 3. Results and Discussion

### 3.1. Physicochemical and Functional Characteristics

Physicochemical and functional characteristics of the different processed cheese formulations are presented in Table 1. The incorporation of walnuts in processed cheeses produced a significant (*p* ≤ 0.05) increase and reduction in % acidity and pH than the control sample, respectively. The acidity increased with the increase in walnut addition. The higher acidity of walnuts in processed cheese could be explained by the acidity of fatty acids present in walnuts and their liberation during cheese processing [11,15].

Moisture content in processed cheeses containing walnuts was significantly (*p* ≤ 0.05) lower than the control, whereas protein, fat, and ash contents followed a reverse trend. Walnuts are rich in nutritional compounds, with high lipid content (55–76%), proteins (11–25%), and carbohydrates (16%) [24]. Therefore, the observed increase in protein, fat, and ash contents is probably due to the composition of added walnut. These results were consistent with other findings in processed cheese with different levels of peanut [11,15] and other dairy products like yoghurt fortified with walnuts [25].

### 3.2. Meltability and Oil Separation

Meltability is one of the most important functional properties of processed cheeses. As shown in Table 1, there were significant differences in the meltability and oil separation index between the control and the processed cheeses containing walnut paste. Data indicated that the meltability of cheese-containing walnut decreased significantly (*p* ≤ 0.05) when increasing the walnuts ratio. Compared with the control, the meltability of cheeses containing walnuts was significantly lower (*p* ≤ 0.05), which means that the control was easily melted when compared to cheeses containing walnut. As previously mentioned, walnut addition affected the compositional profile of processed cheeses, especially dry matter and pH values (Table 1). Those two factors have tremendous importance in the texture, rheological and melting behavior of cheeses, especially through their influence on casein–casein, mineral–casein, and casein–water interactions [26]. These results were in agreement with Awad, Abdel-Hamid, El-Shabrawy and Singh [27], Rafiq and Ghosh [11] and Monteiro, Tavares, Kindstedt and Gigante [28].

Regarding the oil separation index of processed cheese samples, data in Table 1 indicated that control cheese showed the highest value while processed cheese with 15% walnut paste had the lowest one. The oil separation index gradually decreased (*p* ≤ 0.05) with increasing the level of walnuts. This observation could be attributed to the effect of walnuts on the emulsification process in the cheese which leads to low oil leakage from the cheese matrix [27].

### 3.3. Fatty Acids Profile of Walnuts Processed Cheeses

The effect of walnut addition on the fatty acids composition of processed cheeses is presented in Table 2. The fatty acids profile is described by 14 fatty acids, α-Linolenic (C18:3*ω*-3), palmitic acids (C16:0), oleic acid (C18:1) and linoleic acid (C18:2*ω*-6) were the most abundant fatty acids in cheese accounting for approximately 88% of total fatty acids in samples containing walnut. The amounts of all fatty acids increased significantly (*p* ≤ 0.05) with higher walnuts ratios.

In the control samples, palmitic acid (C16:0) was the most abundant fatty acid, followed by myristic (C14:0), and stearic (C18:0) acids. In the cheeses containing walnut paste, linolenic acid (C18:3) was the most abundant fatty acid, followed by oleic (C18:1), palmitic (C16:0), linoleic (C18:2), and stearic (C18:0) acids. C18:1, C18:2, and C18:3 are some of the most vital fatty acids that are necessary to maintain human health [29]. A high improvement in fatty acids (C16:1, C18:1, C18:2, and C18:3) was observed in walnut-added processed cheeses. These findings are consistent with the lipid profile of walnuts, which is rich in PUFAs, with linoleic and linolenic acids accounting for 49–62% and 6–13%, respectively, of total fatty acids [30]. Pereira, Oliveira, Sousa, Ferreira, Bento and Estevinho [2] showed that walnuts have a high content of MUFAs as oleic acid and PUFAs as linolenic and linoleic acids. The fatty acid composition of walnut is 59.7% linoleic acid, 13.1% α-linolenic acid, 15.9% oleic acid, 2.8% stearic, and 8.1% palmitic acid [3]. Thus, fortification with walnut increased MUFAs and PUFAs content of fortified cheeses and improved the *ω*_6_/*ω*_3_ to a more desirable range.

### 3.4. Health-Related Lipids Indices

To evaluate the nutritional value of lipids, the different sum of fatty acids and lipid health indices are presented in Table 3. Compared with control, walnuts processed cheeses presented a larger quantity of MUFAs and PUFAs (mainly α-linolenic acid), a higher (*p* ≤ 0.05) PUFA/SFA ratio, a higher PUFA/MUFA ratio, a higher DFA, and a lower *ω*6/*ω*3 ratio. As walnuts are very rich in MUFAs, and an increase (*p* ≤ 0.05) in the ratio of UFA/SFA was also achieved. The cheeses developed had a low (*p* ≤ 0.05) *ω*6/*ω*3 ratio less than 0.61% compared to 2.06 in control cheese. The highest added walnut amount significantly lowered the *ω*6/*ω*3 of the product (*p* ≤ 0.05). Nutritional guidelines recommend a PUFA/SFA ratio between 0.4–1.0 and *ω*6/*ω*3 PUFAs less than 4 to prevent cardiovascular disease [31].

The atherogenic and thrombogenic indices are interesting parameters that are calculated through the FA profile (Table 2). The atherogenic index (showing the inhibition of the aggregation of plaque) and thrombogenic index (showing the tendency to form clots in the blood vessels) of walnut processed cheeses were significantly lower (*p* ≤ 0.05) than (ranges of 0.80–1.21 and 0.32–0.50, respectively) those of control (6.85 and 4.74, respectively). The obtained remarkable decreases in AI and TI in walnut processed cheeses were very appealing to introduce a high-fat cheese with elevated health scores.

Due to the lowest USFAs (10.37%), control cheese is characterized by the most unfavorable DFA, AI, and TI. On the opposite of this, the walnut-processed cheeses are characterized by the best health lipid indices. Due to the lack of equivalent study on dairy products in the literature, our observations were compared to other studies performed on other food products (beef steak, pork and chicken frankfurters) (Ayo et al. 2007; Cofrades et al. 2004; Nieto et al. 2017; Serrano et al. 2005, 2007) fortified with walnuts and corroborated our present observations. Therefore, the addition of walnuts could be a good strategy to improve the nutritional value of processed cheese and to achieve a wider spectrum of health benefits to the consumers against cardiovascular diseases.

### 3.5. Microstructure of Processed Cheese

**Figure 1:**
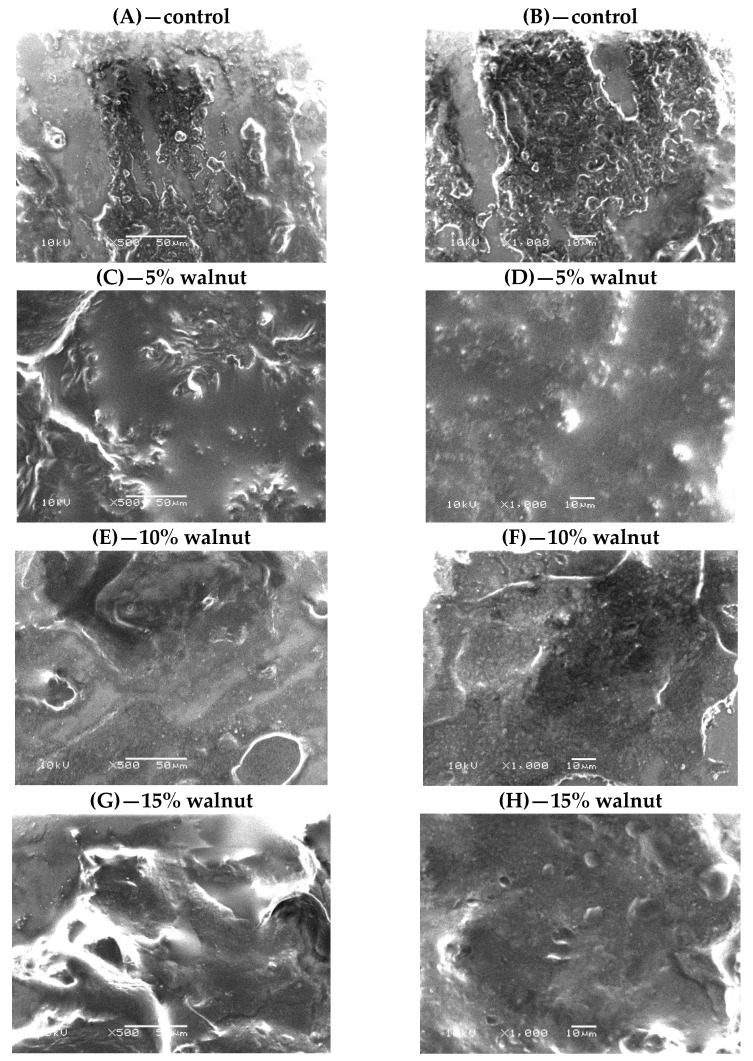
SEM images of processed cheese containing 0% (**A**,**B**); 5% (**C**,**D**); 10% (**E**,**F**) and 15% (**G**,**H**) walnut paste. At different magnitudes 500× (**A**,**C**,**E**,**G**) and 1000× (**B**,**D**,**F**,**H**); at 10 kV; Bar = 50 µm.

Scanning electron micrographs of processed cheese formulated with 0, 5, 10, and 15% walnut pastes are shown in Figure 1. The micrographs of the control (Figure 1A,B) depicted a heterogeneous surface with numerous cavities and pores. A protein matrix enmeshing mainly fat globules can also be distinguished. When adding walnut paste, fat and protein separation was less evidenced (Figure 1C–H). The walnut paste is well incorporated within the protein matrix, producing a compact and continuous surface protein matrix with small-added walnuts pieces dispersed in the protein network. This is due to the effect of walnut fats on the fatty phase of the cheese matrix and the emulsification action of walnut fats [32].

### 3.6. Sensory Evaluation

Table 4 shows the mean scores of appearance, texture, taste, smell, and overall acceptability of control and experimental processed cheeses. All the walnut processed cheeses received high appreciation for the different sensory attributes suggesting that they were well accepted by the panelists. Generally, processed cheeses with 5 or 10% of added walnut presented the highest score for all sensorial attributes compared with cheese containing 15% walnut. Regarding appearance, a significant difference (*p ≤* 0.05) was observed between the cheese control and walnut-added cheeses. For texture, the lowest score was shown with 15% walnut processed cheese. This is probably due to a major reduction in water content, which resulted in a harder and chewier structure. The taste and smell of processed cheese increased with increasing the walnut addition up to the level of 10%, while processed cheese with 15% walnut had scored lower values than other treatments but still better than control cheese. The decrease in taste and smell could be attributed to the over the ratio of walnut and the conflict between walnut and base cheese taste and smell. This might be due to the over intensity of flavour in processed cheese with 15% walnut. These results highlight that there was a balance between the taste and smell of walnut and cheese base in processed cheese with 5% and 10% walnut, which allowed panelists to recognize the pleasant taste of processed cheese with added walnut. The panelists preferred the taste and smell of processed cheese with added 5 or 10% walnut. Similar trends in sensory properties were reported when peanuts were added to processed cheese [11].

### 3.7. Principal Components Analysis

**Figure 2:**
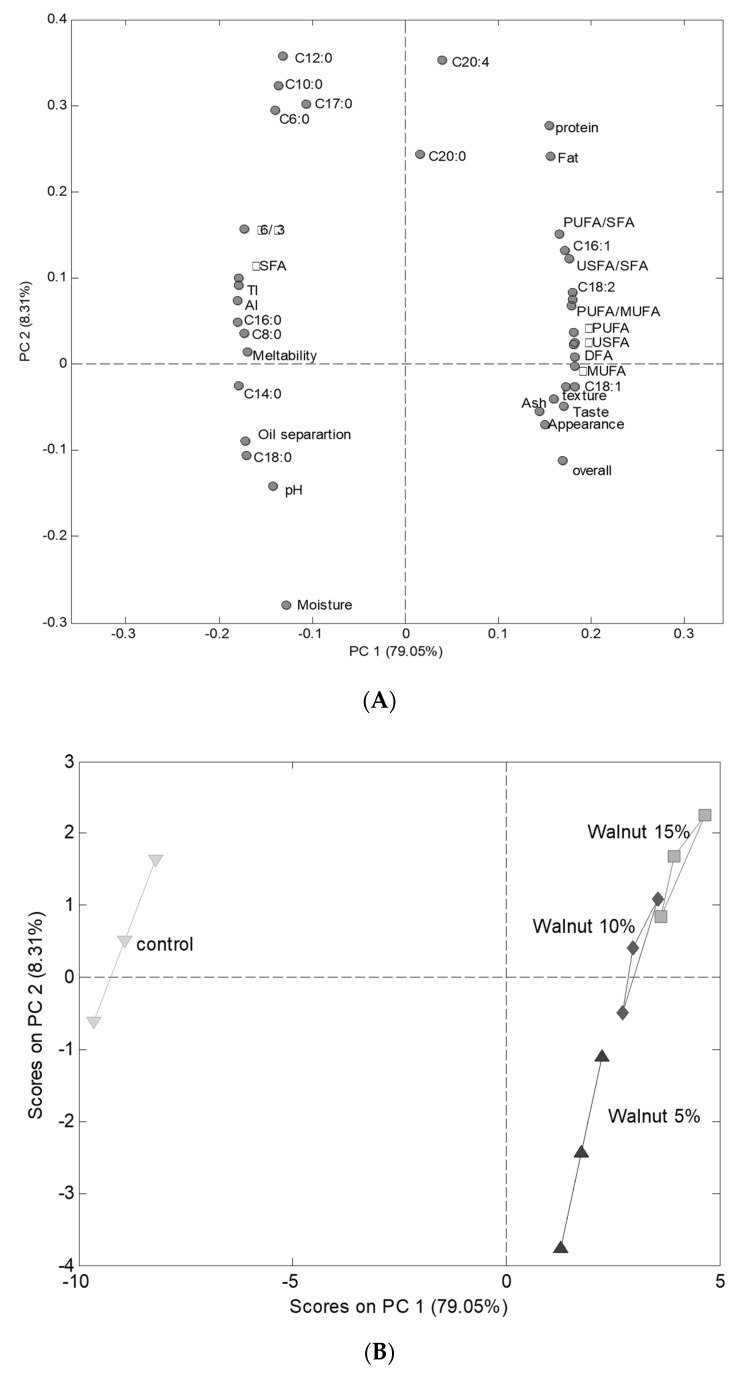
PCA plots (**A**) similarity map and (**B**) loading map obtained by principal components analysis of physicochemical, functional, sensory and fatty acids parameters of processed cheese containing 0, 5, 10, and 15% walnut paste.

Before performing PCA, the datasets obtained from physicochemical, functional and sensory analysis were concatenated in one matrix. This joint analysis was performed in order to go further in the interpretation of the relationship between the measured cheese parameters and to obtain a global overview of the effects of the incorporation of walnut in cheese formulation. Figure 2A,B presented the PCA plots; Figure 2A presented the similarities between cheese samples; Figure 1B exhibited the loading map. The first two principal components accounted for 87.36% of the total variance with a predominance of the principal component 1 (PC_1_) (79.05% of the variance). PC_1_ separated the control cheeses from cheeses containing added walnuts. The control cheeses presented negative scores in the PC_1_ while the other cheese samples presented positive scores. Moreover, the scores values on PC_1_ decreased from cheeses containing higher walnut content to cheeses without walnut.

These observations suggested that PC_1_ discriminated the samples according to their walnut content. The examination of the loading map showed clear differences between cheeses containing walnut and the control cheeses. Different cheese features increased with increasing walnut content. We can mention the acidity, fat, protein and ash contents of cheeses. For fatty acids content, increasing walnut in the cheese formulations increased the C16:1, C18:1, C18:2, C18:3, ΣUSFA, ΣPUFA, PUFA/MUFA ratio, PUFA/SFA ratio and DFA contents. Concerning sensory parameters, increasing walnut content in the cheese formulations increased texture, appearance, smell, taste and overall acceptance. The other measured cheese features were negatively affected by the addition of walnut in the cheese formulation (e.g., pH, meltability, oil separation index). All those conclusions corroborated with previous conclusions mentioned in the present paper.

## 4. Conclusions

In this study, a new processed cheese was developed with the incorporation of walnut paste. The level of walnut paste addition was optimized at 5 or 10% level in processed cheese formulations. The addition of walnut paste in cheese increased PUFA, PUFA/SFA, and *ω*-_6_ values, whereas SFA, TI, AI, and *ω*-_6_/*ω*-_3_ decreased as compared to the control sample. From the Scanning Electron Micrograph of processed cheese, it was observed that walnut paste was uniformly distributed in the protein matrix. Results indicate that walnut paste supplementation can be used as a nutritional strategy to increase concentrations of human health-promoting fatty acids in processed cheeses.

## Figures and Tables

**Table 1:** Physiochemical and functional characteristics of processed cheeses containing 0, 5, 10 and 15% walnut paste.

Attributes	Samples			
	Control	5% Walnuts	10% Walnuts	15% Walnuts
pH	5.20 ± 0.00 ^a^	5.13 ± 0.06 ^ab^	5.07 ± 0.06 ^bc^	5.00 ± 0.00 ^c^
Acidity (%)	1.32 ± 0.08 ^c^	1.87 ± 0.13 ^b^	1.95 ± 0.05 ^b^	2.10 ± 0.02 ^a^
Moisture (%)	40.76 ± 0.06 ^a^	40.63 ± 0.31 ^a^	38.41 ± 0.27 ^b^	38.34 ± 0.07 ^b^
Fat (%)	28.33 ± 0.29 ^d^	29.50 ± 0.00 ^c^	31.67 ± 0.29 ^b^	32.17 ± 0.29 ^a^
Protein (%)	23.03 ± 0.45 ^b^	23.53 ± 0.30 ^b^	24.24 ± 0.06 ^a^	24.45 ± 0.13 ^a^
Ash (%)	4.78 ± 0.07 ^c^	5.02 ± 0.06 ^b^	5.41 ± 0.02 ^a^	5.50 ± 0.10 ^a^
Meltability (%)	38.33 ± 1.44 ^a^	21.67 ± 3.82 ^b^	20.83 ± 2.89 ^c^	14.17 ±1.44 ^d^
Oil separation index (%)	32.47 ± 0.23 ^a^	28.04 ± 0.11 ^b^	26.92 ± 0.35 ^c^	24.66 ± 0.02 ^d^

Results are expressed as mean ± SD; means with different superscripts in a row differ significantly (*p* ≤ 0.05).

**Table 2:** Fatty acids profile of processed cheeses containing 0, 5, 10, and 15% walnut paste.

Fatty Acids	Samples			
	Control	5% Walnuts	10% Walnuts	15% Walnuts
**Saturated fatty acids (SFA %)**				
Caproic acid (C6:0)	3.21 ± 0.27 ^a^	2.41 ± 0.01 ^c^	2.45 ± 0.01 ^c^	2.76 ± 0.02 ^b^
Caprylic acid (C8:0)	4.27 ± 0.30 ^a^	2.87 ± 0.04 ^b^	2.27 ± 0.05 ^d^	2.56 ± 0.03 ^c^
Capric acid (C10:0)	5.91 ± 0.40 ^a^	3.17 ± 0.03 ^d^	3.44 ± 0.06 ^c^	4.70 ± 0.02 ^b^
Lauric acid (C12:0)	6.33 ± 0.37 ^a^	0.95 ± 0.05 ^c^	3.62 ± 0.01 ^b^	3.63 ± 0.01 ^b^
Myristic acid (C14:0)	11.74 ± 0.26 ^a^	6.89 ± 0.02 ^b^	5.75 ± 002 ^c^	4.74 ± 0.04 ^d^
Palmitic acid (C16:0)	16.78 ± 0.25 ^a^	10.04 ± 0.07 ^b^	8.69 ± 0.08 ^d^	9.02 ± 0.05 ^c^
Margaric acid (C17:0)	1.62 ± 0.28 ^a^	0.57 ± 0.09 ^d^	1.37 ± 0.05 ^b^	0.77 ± 0.09 ^c^
Stearic acid (C18:0)	7.82 ± 0.29 ^a^	6.25 ± 0.01 ^b^	5.24 ± 0.06 ^c^	4.82 ± 0.06 ^d^
Arachidic acid (C20:0)	0.10 ± 0.01 ^a^	0.09 ± 0.01 ^a^	0.11 ± 0.01 ^a^	0.12 ± 0.02 ^a^
**Unsaturated fatty acids (USFA %)**				
Palmitoleic acid (C16:1)	4.17 ± 0.12 ^c^	5.49 ± 0.49 ^b^	5.57 ± 0.45 ^b^	6.32 ± 0.12 ^a^
Oleic acid (C18:1)	3.59 ± 0.18 ^d^	8.49 ± 0.35 ^c^	8.64 ± 0.40 ^b^	9.24 ± 0.35 ^a^
Linoleic acid (C18:2) *ω*_6_	1.69 ± 0.45 ^d^	6.51 ± 0.66 ^c^	8.60 ± 0.45 ^b^	9.02 ± 0.66 ^a^
Linolenic acid (C18:3) *ω*_3_	0.82 ± 0.25 ^d^	11.38 ± 0.44 ^c^	13.85 ± 0.45 ^b^	14.61 ± 0.33 ^a^
Arachidonic acid (C20:4)	0.10 ± 0.45 ^c^	0.09 ± 0.22 ^b^	0.08 ± 0.19 ^b^	0.30 ± 0.15 ^a^

Results are expressed as mean ± SD; means with different superscripts in a row differ significantly (*p* ≤ 0.05).

**Table 3:** The ratio of fatty acids and health lipid indices of processed cheeses containing 0, 5, 10, and 15% walnut paste.

Indices	Samples			
	Control	5% Walnuts	10% Walnuts	15% Walnuts
Σ SFA (%)	57.88 ± 0.24 ^a^	33.24 ± 0.25 ^b^	32.76 ± 0.26 ^c^	33.12 ± 0.28 ^b^
Σ USFA (%)	10.37 ± 0.26 ^d^	31.96 ± 0.23 ^c^	36.74 ± 0.14 ^b^	39.49 ± 0.15 ^a^
Σ MUFA (%)	7.76 ± 0.24 ^d^	13.98 ± 0.13 ^c^	14.21 ± 0.16 ^b^	15.56 ± 0.18 ^a^
Σ PUFA (%)	2.61 ± 0.22 ^d^	17.98 ± 0.21 ^c^	22.53 ± 0.12 ^b^	23.93 ± 0.27 ^a^
USFA/SFA ratio	0.18 ± 0.21 ^d^	0.96 ± 0.16 ^c^	1.12 ± 0.11 ^b^	1.31 ± 0.09 ^a^
PUFA/MUFA ratio	0.33 ± 0.09 ^c^	1.28 ± 0.16 ^b^	1.58 ± 0.13 ^a^	1.54 ± 0.13 ^a^
PUFA/SFA ratio	0.04 ± 0.18 ^c^	0.54 ± 0.13 ^b^	0.68 ± 0.15 ^a^	0.72 ± 0.16 ^a^
*ω*_6_/*ω*_3_ ratio	2.06 ± 0.12 ^a^	0.57 ± 0.15 ^b^	0.62 ± 0.09 ^b^	0.61 ± 0.11 ^b^
Desirable fatty acids (DFA) (%)	18.19 ± 0.21 ^d^	38.21 ± 0.22 ^c^	41.98 ± 0.19 ^b^	44.29 ± 0.13 ^a^
Atherogenic index (AI) (%)	6.85 ± 0.01 ^a^	1.21 ± 0.02 ^b^	0.96 ± 0.00 ^c^	0.80 ± 0.01 ^d^
Thrombogenic index (TI) (%)	4.74 ± 0.09 ^a^	0.50 ± 0.08 ^b^	0.36 ± 0.01 ^c^	0.32 ± 0.01 ^c^

Results are expressed as mean ± SD; means with different superscripts in a row differ significantly (*p* ≤ 0.05).

**Table 4:** Sensory characteristics of processed cheeses containing 0, 5, 10, and 15% walnut paste.

Attribute	Samples			
	Control	5% Walnuts	10% Walnuts	15% Walnuts
Appearance	7.00 ± 0.47 ^c^	8.60 ± 0.52 ^a^	8.20 ± 0.63 ^ab^	7.90 ± 0.57 ^b^
Texture	7.20 ± 0.42 ^c^	8.80 ± 0.42 ^a^	8.70 ± 0.48 ^a^	7.90 ± 0.57 ^b^
Taste	6.90 ± 0.32 ^c^	8.80 ± 0.42 ^a^	8.60 ± 0.52 ^a^	8.40 ± 0.52 ^b^
Smell	6.70 ± 0.67 ^c^	8.90 ± 0.32 ^a^	8.80 ± 0.42 ^a^	8.60 ± 0.52 ^b^
Overall acceptability	27.80 ± 0.92 ^c^	35.10 ± 1.20 ^a^	34.30 ± 1.25 ^a^	33.10 ± 1.20 ^b^

Results are expressed as mean ± SD; means with different superscripts in a row differ significantly (*p ≤* 0.05).

## Data Availability

Not applicable.

## References

[1] Jiménez-Colmenero F., Sánchez-Muniz F.J., Olmedilla-Alonso B. (2010). Design and development of meat-based functional foods with walnut: Technological, nutritional and health impact. Food Chem..

[2] Pereira J.A., Oliveira I., Sousa A., Ferreira I.C., Bento A., Estevinho L. (2008). Bioactive properties and chemical composition of six walnut (*Juglans regia* L.) cultivars. Food Chem. Toxicol..

[3] Ros E. (2010). Health benefits of nut consumption. Nutrients.

[4] Moigradean D., Poiana M.-A., Alda L.-M., Gogoasa I. (2013). Quantitative identification of fatty acids from walnut and coconut oils using GC-MS method. Journal of Agroalimentary. Process. Technol..

[5] Grosso G., Estruch R. (2016). Nut consumption and age-related disease. Maturitas.

[6] Petrović-Oggiano G., Debeljak-Martačić J., Ranković S., Pokimica B., Mirić A., Glibetić M., Popović T. (2020). The effect of walnut consumption on n-3 fatty acid profile of healthy people living in a non-Mediterranean West Balkan country, a small-scale randomized study. Nutrients.

[7] Serrano A., Librelotto J., Cofrades S., Sánchez-Muniz F.J., Jiménez-Colmenero F. (2007). Composition and physicochemical characteristics of restructured beef steaks containing walnuts as affected by cooking method. Meat Sci..

[8] Guinee T. (2011). Effects of natural cheese characteristics and processing conditions on rheology and texture: The functionality of cheese components in the manufacture of processed cheese. Process. Cheese Analog..

[9] Solhi P., Azadmard-Damirchi S., Hesari J., Hamishehkar H. (2020). Production of the processed cheese containing tomato powder and evaluation of its rheological, chemical and sensory characteristics. J. Food Sci. Technol..

[10] Solhi P., Azadmard-Damirchi S., Hesari J., Hamishehkar H. (2020). Effect of fortification with asparagus powder on the qualitative properties of processed cheese. Int. J. Dairy Technol..

[11] Rafiq S., Ghosh B. (2017). Effect of Peanut Addition on the Fatty Acid Profile and Rheological Properties of Processed Cheese. Food Process. Technol..

[12] Mohamed A., Abo-El-Khair B., Shalaby S.M. (2013). Quality of novel healthy processed cheese analogue enhanced with marine microalgae Chlorella vulgaris biomass. World Appl. Sci. J..

[13] Krumov K., Ivanov G., Slavchev A., Nenov N. (2010). Improving the processed cheese quality by the addition of natural spice extracts. Adv. J. Food Sci. Technol..

[14] Giri A., Kanawjia S.K., Singh M.P. (2017). Effect of inulin on physico-chemical, sensory, fatty acid profile and microstructure of processed cheese spread. J. Food Sci. Technol..

[15] El-Sayed S.M., Salama H.H., El-Sayed M.M. (2020). Function processed cheese sauce fortified with peanut butter. J. Food Process. Preserv..

[16] Ye A., Cui J., Taneja A., Zhu X., Singh H. (2009). Evaluation of processed cheese fortified with fish oil emulsion. Food Res. Int..

[17] AOAC (2010). Association of Official Analytical Chemists.

[18] Wang F., Zhang X., Luo J., Guo H., Zeng S.S., Ren F. (2011). Effect of Proteolysis and Calcium Equilibrium on Functional Properties of Natural Cheddar Cheese during Ripening and the Resultant Processed Cheese. J. Food Sci..

[19] Thomas M. (1973). The use of a hard milkfat fraction in processed cheese. Aust. J. Dairy Technol..

[20] Maguire L., O’sullivan S., Galvin K., O’connor T., O’brien N. (2004). Fatty acid profile, tocopherol, squalene and phytosterol content of walnuts, almonds, peanuts, hazelnuts and the macadamia nut. Int. J. Food Sci. Nutr..

[21] Ahmad N., Manzoor M.F., Shabbir U., Ahmed S., Ismail T., Saeed F., Nisa M., Anjum F.M., Hussain S. (2020). Health lipid indices and physicochemical properties of dual fortified yogurt with extruded flaxseed omega fatty acids and fibers for hypercholesterolemic subjects. Food Sci. Nutr..

[22] Cunha C.R., Dias A.I., Viotto W.H. (2010). Microstructure, texture, colour and sensory evaluation of a spreadable processed cheese analogue made with vegetable fat. Food Res. Int..

[23] Amini R.K., Islam M.Z., Kitamura Y., Kokawa M. (2019). Utilization of fermented rice milk as a novel coagulant for development of paneer (soft cheese). Foods.

[24] Patraş A., Dorobanţu P. (2010). Physical and chemical composition of some walnut (*Juglans regia* L) biotypes from Moldavia. Lucrări Ştiinţifice.

[25] Ozturkoglu-Budak S., Akal C., Yetisemiyen A. (2016). Effect of dried nut fortification on functional, physicochemical, textural, and microbiological properties of yogurt. J. Dairy Sci..

[26] Lamichhane P., Kelly A.L., Sheehan J.J. (2018). Symposium review: Structure-function relationships in cheese. J. Dairy Sci..

[27] Awad R., Abdel-Hamid L., El-Shabrawy S., Singh R. (2004). Physical and sensory properties of block processed cheese with formulated emulsifying salt mixtures. Int. J. Food Prop..

[28] Monteiro R., Tavares D., Kindstedt P., Gigante M. (2009). Effect of pH on microstructure and characteristics of cream cheese. J. Food Sci..

[29] Goh K.K.T., Ye A., Dale N. (2006). Characterisation of ice cream containing flaxseed oil. Int. J. Food Sci. Technol..

[30] Krauss R.M., Eckel R.H., Howard B., Appel L.J., Daniels S.R., Deckelbaum R.J., Erdman J.W., Kris-Etherton P., Goldberg I.J., Kotchen T.A. (2000). AHA Dietary Guidelines: Revision 2000: A statement for healthcare professionals from the Nutrition Committee of the American Heart Association. Circulation.

[31] Wood J.D., Richardson R.I., Nute G.R., Fisher A.V., Campo M.M., Kasapidou E., Sheard P.R., Enser M. (2004). Effects of fatty acids on meat quality: A review. Meat Sci..

[32] Jiménez Colmenero F., Serrano A., Ayo J., Solas M.T., Cofrades S., Carballo J. (2003). Physicochemical and sensory characteristics of restructured beef steak with added walnuts. Meat Sci..

